# Supplementary material for: Self-Reported Psychosomatic Complaints and Conduct Problems in Swedish Adolescents
Source: Children (Basel). 2022 Jun 27;9(7):963. doi: 10.3390/children9070963 (PMC9324185; doi:10.3390/children9070963)
Supplement: Supplementary file 1 [file children-09-00963-s001.zip › children-1720431-Supplementary.pdf]

Table S1. Questions and scoring from the Uppsala Life and Health Young Cross-sectional Survey (LHS) selected by two independent researchers to represent DSM5 Conduct Disorder (CD) categories.

LHS Question No.	LHS Question (Aggression: people or animals (APA)	LHS Scoring
C5	Have you exposed someone else to bullying, threats or harassment via mobile or SMS?	1=No, 2=One time, 3=Many times
F16	Have you beaten, kicked or exposed someone else to violence during the school term?	1=No, 2=One time, 3=Many times
F18	Have you been annoyed by someone else this semester?	1=No, 2=One time, 3=Many times
G1-12	Have you participated in fights at school?	1=Never, 2=One time, 3=2-4 times, 4=5-10 times, 5=More than 10 times
G1-13	Have you illegally carried a knife or other weapon in school?	1=Never, 2=One time, 3=2-4 times, 4=5-10 times, 5=More than 10 times
G1-14	Have you illegally carried a knife or other weapon in the town?	1=Never, 2=One time, 3=2-4 times, 4=5-10 times, 5=More than 10 times
G1-15	Have you had sex with someone against their will?	1=Never, 2=One time, 3=2-4 times, 4=5-10 times, 5=More than 10 times
G1-16	Have you failed to make a payment at the cinema, cafe, train or bus?	1=Never, 2=One time, 3=2-4 times, 4=5-10 times, 5=More than 10 times
G1-17	Have you threatened or forced someone to do something they didn't want to do?	1=Never, 2=One time, 3=2-4 times, 4=5-10 times, 5=More than 10 times
G1-18	Have you driven a moped, car or motorcycle while drinking alcohol?	1=Never, 2=One time, 3=2-4 times, 4=5-10 times, 5=More than 10 times
G1-19	Did you ever get stopped by the police for something you did?	1=Never, 2=One time, 3=2-4 times, 4=5-10 times, 5=More than 10 times<br>**Min Score: 11, Max Score: 49**

LHS Question No.	LHS Question (Destruction of property (DP))	LHS Scoring
G1-10	Have you deliberately destroyed or been involved in destroying things?	1=Never, 2=One time, 3=2-4 times, 4=5-10 times, 5=More than 10 times
G1-11	Have you been involved in scribbling, graffiti painting or writing something with a marker or spray paint without permission?	1=Never, 2=One time, 3=2-4 times, 4=5-10 times, 5=More than 10 times<br>**Min Score: 2; Max Score: 10**

LHS Question No.	LHS Question (Deceitfulness or theft (DT))	LHS Scoring

G1-1	Do you have or had gambling debts to others?	1=Never, 2=One time, 3=2-4 times, 4=5-10 times, 5=More than 10 times
G1-2	Have you taken goods from a department store, kiosk or store without paying?	1=Never, 2=One time, 3=2-4 times, 4=5-10 times, 5=More than 10 times
G1-3	Have you taken money at home that isn't yours?	1=Never, 2=One time, 3=2-4 times, 4=5-10 times, 5=More than 10 times
G1-4	Have you stolen money or things from any schoolmate?	1=Never, 2=One time, 3=2-4 times, 4=5-10 times, 5=More than 10 times
G1-5	Have you threatened or forced someone to give you money, cell phone, cigarettes or anything else?	1=Never, 2=One time, 3=2-4 times, 4=5-10 times, 5=More than 10 times
G1-6	Have you been involved in making a burglary?	1=Never, 2=One time, 3=2-4 times, 4=5-10 times, 5=More than 10 times
G1-7	Have you stolen a bike?	1=Never, 2=One time, 3=2-4 times, 4=5-10 times, 5=More than 10 times
G1-8	Have you stolen a car?	1=Never, 2=One time, 3=2-4 times, 4=5-10 times, 5=More than 10 times
G1-9	Have you sold or bought something that you knew was stolen?	1=Never, 2=One time, 3=2-4 times, 4=5-10 times, 5=More than 10 times<br>**Min Score: 9; Max Score: 45**

LHS Question No.	LHS Question (Serious violation of rules (SVR))	LHS Scoring
A10-1	How easy is it for you at home to say where you go?	1=Many times, 2=sometimes, 3=not so many times, 4=Never
A10-2	How easy is it for you to work on your schoolwork at home?	1=Many times, 2=sometimes, 3=not so many times, 4=Never
A10-3	How easy is it for you to go home?	1=Many times, 2=sometimes, 3=not so many times, 4=Never
A10-4	How easy is it for you to feel close to your home?	1=Many times, 2=sometimes, 3=not so many times, 4=Never
A10-5	How easy is it for you to sleep at home?	1=Many times, 2=sometimes, 3=not so many times, 4=Never
A10-6	How easy is it for you help at home?	1=Many times, 2=sometimes, 3=not so many times, 4=Never
A10-7	How easy is it for you to be at home when you are not at school?	1=Many times, 2=sometimes, 3=not so many times, 4=Never
F12	Are you truant from school?	1=No never, 2=yes, once in the term, 3=yes, once in the month, 4=yes, 2-3 times in the month, 5=yes, once

		a week, 6=yes, more than once a week
F13	Have you not attended some of your subjects?	1=No, not in any subject, 2=Yes, 1-2 subjects, 3=Yes, 3-4 subjects, 4=Yes, 5 or more subjects
F14	Have you felt uncomfortable because of fear of falling into trouble during the school day?	1=No, 2=Yes one time, 3=yes, more than one time
		**Min Score: 10; Max Score: 41**
		**Total CD Min score:32; Max Score: 145**

(NB:High score denotes high level of self-reported behavioural issues); CD=Conduct Disorder; Min=Minimum; Max=Maximum; LHS=Life Health Survey Questionnaire

Table S2. Questions and scoring from the Uppsala Life and Health Young Cross-sectional Survey (LHS) selected by two independent researchers to represent DSM5 Conduct Disorder (CD) categories.

LHS Question No.	LHS Question (Aggression: people or animals (APA)	LHS Scoring
C5	Have you exposed someone else to bullying, threats or harassment via mobile or SMS?	1=No, 2=One time, 3=Many times
F16	Have you beaten, kicked or exposed someone else to violence during the school term?	1=No, 2=One time, 3=Many times
F18	Have you been annoyed by someone else this semester?	1=No, 2=One time, 3=Many times
G1-12	Have you participated in fights at school?	1=Never, 2=One time, 3=2-4 times, 4=5-10 times, 5=More than 10 times
G1-13	Have you illegally carried a knife or other weapon in school?	1=Never, 2=One time, 3=2-4 times, 4=5-10 times, 5=More than 10 times
G1-14	Have you illegally carried a knife or other weapon in the town?	1=Never, 2=One time, 3=2-4 times, 4=5-10 times, 5=More than 10 times
G1-15	Have you had sex with someone against their will?	1=Never, 2=One time, 3=2-4 times, 4=5-10 times, 5=More than 10 times
G1-16	Have you failed to make a payment at the cinema, cafe, train or bus?	1=Never, 2=One time, 3=2-4 times, 4=5-10 times, 5=More than 10 times
G1-17	Have you threatened or forced someone to do something they didn't want to do?	1=Never, 2=One time, 3=2-4 times, 4=5-10 times, 5=More than 10 times
G1-18	Have you driven a moped, car or motorcycle while drinking alcohol?	1=Never, 2=One time, 3=2-4 times, 4=5-10 times, 5=More than 10 times
G1-19	Did you ever get stopped by the police for something you did?	1=Never, 2=One time, 3=2-4 times, 4=5-10 times, 5=More than 10 times
		**Min Score: 11, Max Score: 49**

LHS Question No.	LHS Question (Destruction of property (DP))	LHS Scoring
G1-10	Have you deliberately destroyed or been involved in destroying things?	1=Never, 2=One time, 3=2-4 times, 4=5-10 times, 5=More than 10 times
G1-11	Have you been involved in scribbling, graffiti painting or writing something with a marker or spray paint without permission?	1=Never, 2=One time, 3=2-4 times, 4=5-10 times, 5=More than 10 times
		**Min Score: 2; Max Score: 10**

LHS Question No.	LHS Question (Deceitfulness or theft (DT))	LHS Scoring
G1-1	Do you have or had gambling debts to others?	1=Never, 2=One time, 3=2-4 times, 4=5-10 times, 5=More than 10 times
G1-2	Have you taken goods from a department store, kiosk or store without paying?	1=Never, 2=One time, 3=2-4 times, 4=5-10 times, 5=More than 10 times
G1-3	Have you taken money at home that isn't yours?	1=Never, 2=One time, 3=2-4 times, 4=5-10 times, 5=More than 10 times
G1-4	Have you stolen money or things from any schoolmate?	1=Never, 2=One time, 3=2-4 times, 4=5-10 times, 5=More than 10 times
G1-5	Have you threatened or forced someone to give you money, cell phone, cigarettes or anything else?	1=Never, 2=One time, 3=2-4 times, 4=5-10 times, 5=More than 10 times
G1-6	Have you been involved in making a burglary?	1=Never, 2=One time, 3=2-4 times, 4=5-10 times, 5=More than 10 times
G1-7	Have you stolen a bike?	1=Never, 2=One time, 3=2-4 times, 4=5-10 times, 5=More than 10 times
G1-8	Have you stolen a car?	1=Never, 2=One time, 3=2-4 times, 4=5-10 times, 5=More than 10 times
G1-9	Have you sold or bought something that you knew was stolen?	1=Never, 2=One time, 3=2-4 times, 4=5-10 times, 5=More than 10 times
		**Min Score: 9; Max Score: 45**

LHS Question No.	LHS Question (Serious violation of rules (SVR))	LHS Scoring
A10-1	How easy is it for you at home to say where you go?	1=Many times, 2=sometimes, 3=not so many times, 4=Never
A10-2	How easy is it for you to work on your schoolwork at home?	1=Many times, 2=sometimes, 3=not so many times, 4=Never

A10-3	How easy is it for you to go home?	1=Many times, 2=sometimes, 3=not so many times, 4=Never
A10-4	How easy is it for you to feel close to your home?	1=Many times, 2=sometimes, 3=not so many times, 4=Never
A10-5	How easy is it for you to sleep at home?	1=Many times, 2=sometimes, 3=not so many times, 4=Never
A10-6	How easy is it for you help at home?	1=Many times, 2=sometimes, 3=not so many times, 4=Never
A10-7	How easy is it for you to be at home when you are not at school?	1=Many times, 2=sometimes, 3=not so many times, 4=Never
F12	Are you truant from school?	1=No never, 2=yes, once in the term, 3=yes, once in the month, 4=yes, 2-3 times in the month, 5=yes, once a week, 6=yes, more than once a week
F13	Have you not attended some of your subjects?	1=No, not in any subject, 2=Yes, 1-2 subjects, 3=Yes, 3-4 subjects, 4=Yes, 5 or more subjects
F14	Have you felt uncomfortable because of fear of falling into trouble during the school day?	1=No, 2=Yes one time, 3=yes, more than one time
		**Min Score: 10; Max Score: 41**
		**Total CD Min score:32; Max Score: 145**

(NB:High score denotes high level of self-reported behavioural issues); CD=Conduct Disorder; Min=Minimum; Max=Maximum; LHS=Life Health Survey Questionnaire

Table S3. LHS scores categorised (by two independent researchers) according to DSM5 CD sub- categories, in terms of low, medium and high scores of self-reported PSC in younger, older and total male and female adolescents (in the total n=3,132 cohort, after exclusions).

Mean%.
[s.d.]

	Low PSC						Medium PSC						High PSC					
	Male			Female			Male			Female			Male			Female		
	Young (n=376)	Older (n=344)	Total (n=720)	Young (n=210)	Older (n=171]	Total (n=381)	Young (n=212)	Older (n=223)	Total (n=435)	Young (n=240)	Older (n=238)	Total (n=478)	Young (n=137)	Older (n=168)	Total (n=305)	Young (n=358)	Older (n=455)	Total (n=813)
APA	**30.29**	**29.79**	**30.05**	**28.16**	**28.49**	**28.31**	**31.14**	**31.28**	**31.21**	**29.41**	**29.25**	**29.33**	**34.90**	**33.83**	**34.31**	**33.04**	**31.34**	**32.09**
	[6.34]	[4.76]	[5.64]	[2.30]	[2.61]	[2.45]	[5.85]]	[5.06]	[5.45]	[3.28]	[2.84]	[3.07]	[10.95]	[7.79]	[9.34]	[7.43]	[4.69]	[6.11]
DT	**24.93**	**25.61**	**25.26**	**22.85**	**23.00**	**22.92**	**25.95**	**26.54**	**26.25**	**23.99**	**23.73**	**23.86**	**28.40**	**28.53**	**28.47**	**26.98**	**26.15**	**26.51**
	[10.25]	[7.61]	[9.083]	[4.59]	[4.45]	[4.52]	[7.93]	[8.50]	[8.23]	[5.14]	[4.92]	[5.03]	[14.47]	[11.78]	[13.03]	[8.69]	[6.67]	[7.63]

DP	**27.23**	**27.47**	**27.35**	**23.10**	**23.27**	**23.18**	**29.34**	**30.00**	**29.68**	**25.42**	**24.75**	**25.08**	**31.82**	**31.90**	**31.87**	**29.80**	**26.73**	**28.08**
	[15.76]	[14.62]	[15.21]	[9.41]	[9.57]	[9.47]	[16.28]	[16.28]	[16.26]	[11.85]	[10.22]	[11.06]	[20.19]	[19.36]	[19.71]	[16.42]	[13.23]	[14.79]
SVR	**42.29**	**44.98**	**43.58**	**38.27**	**42.66**	**40.24**	**42.96**	**48.44**	**45.77**	**39.91**	**43.72**	**41.81**	**46.10**	**50.01**	**48.26**	**44.92**	**47.50**	**46.37**
	[7.72]	[8.05]	[7.99]	[6.67]	[7.69]	[7.46]	[7.84]	[9.25]	[9.01]	[7.06]	[8.30]	[7.93]	[10.35]	[10.28]	[10.48]	[9.69]	[9.34]	[9.58]
**Total**	**31.70**	**32.33**	**32.00**	**28.99**	**30.27**	**29.57**	**32.61**	**34.22**	**33.44**	**30.38**	**31.14**	**30.76**	**35.81**	**36.35**	**36.11**	**34.22**	**33.73**	**33.94**
**CD**	[6.79]	[5.28]	[6.12]	[3.13]	[3.49]	[3.35]	[5.82]	[5.60]	[5.76]	[3.77]	[3.71]	[3.75]	[10.36]	[8.23]	[9.24]	[7.26]	[5.30]	[6.24]

APA=Aggression to people or animals; DP=Destruction of property; DT=Deception or theft; SVR=Serious violation of rules; N=Number of participants per sub-sample – missing values excluded; s.d.=standard deviation; young=15-16yrs; older=17-19yrs; PSC=Psychosomatic Complaints; Low PSC=0-33 percentile, Medium PSC=34-66 percentile, High PSC=67-100 percentile
